# Comparison of testing methods assessing the in vitro efficacy of the combination of aztreonam with avibactam on multidrug-resistant Gram-negative bacilli

**DOI:** 10.1186/s12941-024-00708-0

**Published:** 2024-05-25

**Authors:** Corentin Deckers, Florian Bélik, Olivier Denis, Pierre Bogaerts, Isabel Montesinos, Catherine Berhin, Warda Bouchahrouf, Martin Hoebeke, Stephanie Evrard, Nicolas Gilliard, Merve Okur, Te-Din Huang

**Affiliations:** National Reference Center for Antibiotic-Resistant Gram-Negative Bacilli, CHU UCL Namur and Université Catholique de Louvain, Avenue Gaston Therasse, 1, 5530 Yvoir, Belgium

**Keywords:** Antibiotic combination, Carbapenemase, Metallo-β-lactamase, Antibiotic susceptibility testing, Aztreonam-avibactam

## Abstract

**Background:**

Aztreonam-avibactam (ATM-AVI) combination shows promising effectiveness on most carbapenemase-producing Gram-negatives, yet standardized antibiotic susceptibility testing (AST) methods for evaluating the combination in clinical laboratories is lacking. We aimed to evaluate different ATM-AVI AST approaches.

**Methods:**

96 characterized carbapenem-resistant clinical isolates belonging to 9 *Enterobacterales* (EB; n = 80) and *P. aeruginosa* (PA; n = 16) species, including 90 carbapenemase producers and 72 strains resistant to both CAZ-AVI and ATM, were tested. Paper disk elution (DE; Bio-Rad) and E-test gradient strips stacking (SS; bioMérieux) were performed for the ATM + CAZ-AVI combination. MIC Test Strip (MTS; Liofilchem) was evaluated for ATM-AVI MIC determination. Results were interpreted applying ATM clinical breakpoints of the EUCAST guidelines and compared to the broth microdilution method (Sensititre, Thermofisher).

**Results:**

According to broth microdilution method, 93% of EB and 69% of PA were tested susceptible to ATM-AVI. The synergistic effect of ATM-AVI was of 95% for EB, but of only 17% for PA. The MTS method yielded higher categorical and essential agreement (CA/EA) rates for both EB (89%/91%) and PA (94%/94%) compared to SS, where the rates were 87%/83% for EB and 81%/81% for PA. MTS and SS yielded 2 and 3 major discrepancies, respectively, while 3 very major discrepancies each were observed for both methods. Concerning the DE method, CA reached 91% for EB and 81% for PA, but high number of very major discrepancies were observed for EB (n = 6; 8%) and for PA (n = 3; 19%).

**Conclusions:**

The ATM-AVI association displayed excellent in vitro activity against highly resistant clinical *Enterobacterales* strains. MTS method offers accurate ATM-AVI AST results, while the SS method might serve as better alternative then DE method in assessing the efficacy of ATM + CAZ-AVI combination. However, further investigation is needed to confirm the methods' ability to detect ATM-AVI resistance.

**Supplementary Information:**

The online version contains supplementary material available at 10.1186/s12941-024-00708-0.

## Introduction and objectives

Multidrug resistance (MDR) in Gram-negative rods represents a major public health issue and epidemiological data indicated a significant increase in the prevalence of carbapenem resistance in Europe and worldwide. Severe infections such as bacteremia caused by carbapenemase-producing organisms (CPO) are of major concern in clinical practice due to the limited therapeutic options available with negative impacting on the outcome of infected patients.

The development of new antimicrobial treatments includes the pairing of modern beta-lactamase inhibitors with traditional beta-lactam agents. Avibactam (AVI), combined with ceftazidime (CAZ) in pharmaceutical available therapy, demonstrates in-vitro and clinical efficacy against several Ambler class A (ESBLs, KPC), C (AmpC) and D (OXA-48) beta-lactamases including carbapenemases, but fails to exhibit efficacy against *Enterobacterales* that express class B metallo-beta-lactamases (MBL) such as NDM, VIM or IMP, that are capable of hydrolyzing carbapenems, often in conjunction with other β-lactams. On the other hand, aztreonam (ATM), a monobactam beta-lactam, has potent activity against MBLs, but the co-expression in MBL-producing *Enterobacterales* of other large spectrum aztreonam-hydrolyzing beta-lactamases like ESBLs and/or AmpC cephalosporinases has made the monotherapy use of ATM difficult.

Clinical trials of investigational antibiotic combination aztreonam-avibactam (ATM-AVI) showed promising results from two phase 3 studies [1, 2]. Meanwhile, the combination of ceftazidime-avibactam (CAZ-AVI) and ATM has demonstrated efficacy against MBL-producing *Enterobacterales* and *Pseudomonas aeruginosa* and could serve as a therapeutic option awaiting the potential approval and release of ATM-AVI for clinical use [3–8]. However, published literature mainly comprises limited clinical experiences through small case series [9].

The latest treatment guidelines issued by European Society of Clinical Microbiology and Infectious Diseases (ESCMID) conditionally recommend the combination of ATM and CAZ-AVI (ATM + CAZ-AVI) for the treatment of patients with severe infections caused by carbapenem-resistant *Enterobacterales* (CRE) harboring MBLs and/or showing resistance to all available antibiotics, especially when the strain is also cefiderocol-resistant [10]. However, a practical and standardized antibiotic susceptibility testing (AST) method for evaluating the efficacy of the ATM + CAZ-AVI combination in clinical laboratories is currently lacking.

This study aimed to assess the performance of various methods for the AST of the ATM + CAZ-AVI combination and of a commercial ATM-AVI gradient strip diffusion method using a collection from the Belgian National Reference Center for Antibiotic-Resistant Gram-Negative Bacilli (NRC) of CRE and MDR *P. aeruginosa*, including a majority of carbapenemase producers.

## Materials and methods

This study panel included 96 previously characterized non-duplicate carbapenem-resistant clinical isolates belonging to 9 *Enterobacterales* and 1 non-fermentative (*P. aeruginosa*) species which are summarized in Table 1. Phenotypically and/or genotypically defined resistance mechanisms to beta-lactams are detailed in Supplementary data S1. The study panel included 70 strains that were resistant to both CAZ-AVI and ATM, 8 strains that were sensitive to CAZ-AVI but resistant to ATM, and 18 strains that were resistant to CAZ-AVI but sensitive to ATM as determined by Sensititre broth microdilution (BMD) assays. Furthermore, this panel included 14 strains exhibiting resistance to cefiderocol by BMD. Such selection was intended to challenge the efficacy of the ATM + CAZ-AVI combination against a collection of MDR strains widely resistant to last-line molecules.

**Table 1:** Characteristics of clinical isolates (n = 96) tested in the study

Species	Number of strains	Mechanisms of resistance to carbapenems	Susceptiblity testing results	
			ATM-R CAZ-AVI-R	Sensititre Cefiderocol-R
*K.pneumoniae*	33	NDM (n = 16), NDM + OXA-48-like (n = 10), VIM (n = 3), KPC (n = 1), IMP + OXA-48-like (n = 1), KPC + OXA-48-like (n = 1), NDM + KPC (n = 1)	28	3
*E. coli*	19	NDM (n = 8), VIM (n = 5), NDM + OXA-48-like (n = 2), IMP (n = 1), KPC (n = 1), OXA-48-like (n = 1), non-carbapenemase (n = 1)	13	5
*C. freundii*	8	VIM (n = 3), NDM + OXA-48-like (n = 2), KPC (n = 1),NDM (n = 1), VIM + OXA-48-like (n = 1)	6	2
*K. oxytoca*	7	NDM (n = 2), VIM (n = 2), KPC (n = 1),NDM + OXA-48-like (n = 1), VIM + KPC (n = 1)	2	2
*E. cloacae*	5	NDM (n = 2), VIM (n = 2), OXA-48-like (n = 1)	4	1
*S. marcescens*	5	NDM (n = 2), IMP (n = 1), OXA-427 (n = 1), non-carbapenemase (n = 1)	3	1
*K. aerogenes*	1	NDM (n = 1)	1	0
*P. mirabilis*	1	NDM (n = 1)	1	0
*P. stuartii*	1	NDM (n = 1)	0	0
Subtotal *Enterobacterales*	80		5	1
*P. aeruginosa*	16	VIM (n = 5), non-carbapenamase (n = 4),, IMP (n = 2), GES-5 (n = 1), NDM (n = 1), NDM + VIM (n = 1)	12	0
Total	96		70	14

ATM: aztreonam; CAZ-AVI: ceftazidim-avibactam; S: susceptible; R: resistant; IMP: imipenem-hydrolyzing metallo-β-lactamase; NDM: New Delhi metallo-β-lactamase; VIM: Verona integron-encoded metallo-β-lactamase; KPC: Klebsiella pneumoniae carbapenemase; GES: Guiana extended-spectrum β-lactamase

All 96 clinical strains were tested using freshly prepared overnight subcultures on 5% sheep blood trypti-soya agar plates. Species identification was carried out using matrix-assisted laser desorption ionization time-of-flight mass spectrometry (MALDI-TOF MS) with MALDI Biotyper (Bruker, Germany). The evaluated AST methods (once per method per strain) included disk elution (DE), gradient diffusion strip stacking (SS), and MIC Test Strip aztreonam-avibactam (MTS; Liofilchem). Reference MIC and category results for ATM-AVI were defined by BMD using Sensititre (MIC range 0,03/4–64/4 μg/mL) (Thermo Fisher Scientific, Waltham, MA, USA). To ensure the reproducibility of the methods employed, three selected MBL-producing positive control strains underwent five repetitions of DE, SS, and MTS in conjunction with BMD for ATM-AVI. In case of an invalid result, the strain was retested with the same method.

Combination disk elution (DE) was performed using CAZ-AVI 10/4 µg disk and ATM 30 µg disk (Bio-Rad Hercules, CA, USA) as described previously [11]. Briefly, a 0,5 McFarland suspension was prepared and 12 µL of this suspension were inoculated onto three separate tubes containing 2 mL of Mueller–Hinton broth (Thermo Fisher Scientific, Waltham, MA, USA). Afterwards, one ATM disk was added to the first tube (theoretical concentration of ATM of 15 µg/mL), two CAZ-AVI disks were added to the second tube (concentration of CAZ-AVI of 10/4 µg/mL), one ATM and two CAZ-AVI disks were added to the third tube (concentration of ATM 15 µg/mL and CAZ-AVI 10/4 µg/mL). After 30-min incubation at room temperature, antibiotics disks were removed, then the tubes were vortexed and incubated for 24 h at 37 °C. The presence or absence of bacterial turbid growth were observed following the incubation by two readers. The effectiveness of the ATM + CAZ-AVI combination was determined by the absence of any visible growth in the tube containing both ATM and CAZ-AVI.

Gradient diffusion strip stacking (SS) method was accomplished by combining the use of E-test strips for CAZ-AVI (MIC range 0,016/4–256/4 μg/mL) and for ATM (MIC range 0,016–256 μg/mL) (bioMérieux, Marcy l’Etoile, France). In this approach, the E-test strip of CAZ-AVI was placed on the agar surface for 10 min. Subsequently, the CAZ-AVI E-test strip was removed, and an ATM E-test strip was positioned at the same spot. After an incubation of 18 h, the MIC was read [11].

MIC Test Strip aztreonam-avibactam (MIC range 0,016/4–256/4 μg/mL) (MTS™, Liofilchem®) was performed according to manufacturers’ instructions and evaluated for the determination of ATM-AVI MIC.

Recorded raw results were interpreted according to the EUCAST 2023 clinical breakpoints, applying the established ATM criteria to assess the ATM-AVI combination as previously performed in other studies (Table 2) [12–14].

**Table 2:** MIC breakpoints for antimicrobials used in the study

Antimicrobials	MIC breakpoints for *Enterobacterales* (mg/L)		MIC breakpoints for *P. aeruginosa* (mg/L)	
	S	R	S	R
Aztreonam (ATM)	≤ 1	> 4	≤ 0,001	> 16
Ceftazidime-avibactam (CAZ-AVI)	≤ 8	> 8	≤ 8	> 8
Aztreonam-avibactam (ATM-AVI)*	≤ 1	> 4	≤ 0,001	> 16

^*^Interpretations for aztreonam-avibactam combination were based on aztreonam EUCASt clinical breaktpoints

ATM: aztreonam; ATM-AVI: aztreonam-avibactam; CAZ-AVI: ceftazidime-avibactam; S: susceptible; R: resistant

The MIC and category results obtained by different methods were compared to the BMD results. Categorical agreement (CA: agreement of category results), essential agreement (EA: MICs within ± 1 dilution of reference MICs, adapted to the range of the tested dilutions by excluding all extreme values of ≤ X and > Y mg/L), very major discrepancy (VMD: false drug-sensitive result), major discrepancy (MD: false drug-resistant result) and minor discrepancy (minD: susceptible by the evaluated routine method versus susceptible at high dose by the reference method or vice-versa) rates were calculated for each method compared to the reference BMD. All methods were evaluated according to the ISO Standard 20776-2 criteria (EA and CA > 90%, VMD < 3%). Regarding the MIC-based methods, activity synergy was defined as a reduction of at least 3 dilutions in the MIC comparing the lowest MIC obtained for either CAZ-AVI or ATM, with MIC of the combination (ATM-AVI). For DE method, synergy was defined as the total growth inhibition when CAZ-AVI and ATM were combined, specifically for strains resistant to CAZ-AVI and ATM separately (CAZ-AVI R, ATM R).

## Results

### Reproducibility

Five results per strain were obtained for reproducibility testing per method. Reproducibility was perfect for all methods (100%) for *E. coli* and *K. pneumoniae* isolates. Regarding *P. aeruginosa* strain, reproducibility reached 100% except for DE method and SS method which were only 80%. (Supplementary data Table 2).

### Method comparison on clinical collection strains

Out of 80 *Enterobacterales* strains tested with ATM-AVI BMD, 66 (82,5%) were susceptible, 8 (10,0%) were susceptible at increased exposure and 6 (7,5%) were resistant to the combination. ATM-AVI resistant strains were 4 *E. coli* (one producing NDM-4 with CTX-M-15 and CMY-6, one NDM-7 with CTX-M-15, one NDM-1 with PER-3, CMY-6 and DHA-1, and one NDM-5 with CMY-42) showing MIC range of 8 to 16 mg/L and 2 *S. marcescens* (one OXA-427 and one non-carbapenemase hyperproducing cephalosporinase) having both a MIC of 16 mg/L. Among the 66 *Enterobacterales* strains resistant to both CAZ-AVI and ATM separately, 93% (n = 62/66) exhibited restored susceptibility with ATM-AVI.

Out of 16 *P. aeruginosa* strains tested with ATM-AVI BMD, 11 (68,7%) were considered susceptible (MIC of 8–16 mg/L) and 5 (31,3%) were resistant (MIC of 64 mg/L) to the combination using the EUCAST ATM clinical resistance breakpoint of > 16 mg/L. ATM-AVI resistant strains were 3 non-carbapenemase strains, one VIM-2 producer and one strain co-producing NDM-1 and VIM-5. Among *P. aerguinosa* strains resistant to CAZ-AVI and to ATM separately, 58% (n = 7/12) had susceptibility restored with ATM-AVI.

The synergistic activity (defined as threefold MIC reduction) with the ATM-AVI combination was observed for 87,5% (n = 70/80) of *Enterobacterales* (including 95% (n = 63/66) that were resistant to both CAZ-AVI and ATM) and only 12,5% (n = 2/16) of *P. aeruginosa* strains (representing one-third of the MBL producers)*.*

### MIC-based methods

A total of 96 organism-drug results per method were obtained to calculate categorization performance (CA, VMD, MD, minD) for MTS, and SS methods. No invalid results were observed by any of the testing methods. Due to truncations in the concentration range of the evaluated method and/or of the reference method, the numbers of evaluable organism-drug results were lower for the calculation of EA (87 for MTS and SS methods). All agreement and discrepancy rates for MIC based methods are detailed in Table 3.

**Table 3:**
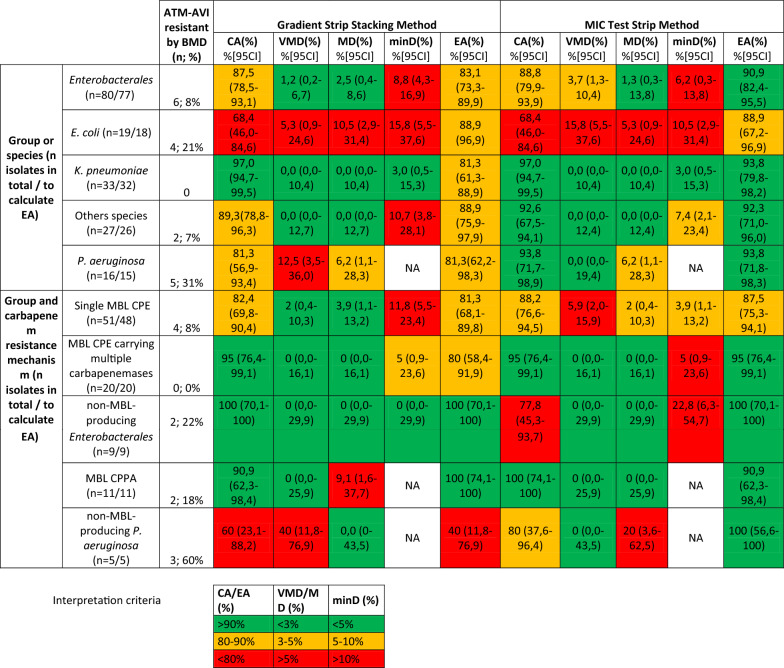
Performances of MIC-based avibactam-aztreonam testing methods

EC: % [95 CI]: Confidence Interval of 95%; AVI-AZT : avibactam-aztreonam; CA: Categorical agreement; EA: essential agreement; MD: Major discrepancy; minD: minor discrepancy; VMD: Very Major Discrepancy; NA: non applicable; BMD: broth microdilution; CPE: carbapenemase-producing enterobacterales; CPPA: carbapenemase-producing pseudomonas aeruginosa; MBL:metallo-beta-lactamase

The MTS method demonstrated higher CA and EA for both *Enterobacterales* (88,8% and 90,9%) and *P. aeruginosa* (93,8% and 93,8%) than SS method (*Enterobacterales* 87,5% and 83,1%, *P. aeruginosa* 81,3% and 88,9%)). This method resulted in 3,7% VMD, 1,3% MD, and 6,2% minD for *Enterobacterales* and 0% VMD and 6,2% MD for *P. aeruginosa. E. coli* species displayed higher rate of VMD, MD and minD among *Enterobacterales* with 15,8%, 5,3% and 10,5% respectively. All other subspecies have a higher CA and EA for MTS than for SS method. Overall, the MTS method yielded 2 MD and 3 VMD. All VMD came from *E. coli* ATM-AVI resistant strains (one NDM-4, CTX-M-15, CMY-6, one NDM-7, CTX-M-15 and one NDM-5 CMY-42 strains) while the 2 MD came from 1 *E. coli* strain (one NDM-5, TEM-187 and CMY-42 strain) and 1 *P. aerguinosa* (GES-5).

Regarding the SS method, we observed a CA and an EA of 87,5% and 83,1% respectively for *Enterobacterales* and 81,3% and 81,3% for *P. aeruginosa*. 1,2% of VMD, 2,5% of MD and 8,8% of minD were found for *Enterobacterales. E. coli* showed the highest rate of minD with 15,8%. No VMD or MD were found for other *Enterobacterales*. Regarding *P. aeruginosa*, 12,5% of VMD and 6,3% of MD were found. Overall, the SS method yielded 3 MD and 3 VMD. Similarly to the MTS method, 1 VMD came from one NDM-7-producing *E. coli* strain while the two other VMD came from 2 strains of non-carbapenemase *P. aeruginosa*. The 3 MD came from a MBL (IMP-1, VEB-1)-producing *P. aeruginosa* and from 2 NDM-producing *E. coli* (1 NDM-5 TEM-187 and CMY-42 strain and 1 NDM-5 and CMY-42 strain).

MIC of ATM-AVI using MTS and SS compared with BMD are shown in Table 4. For *Enterobacterales,* MIC values obtained with the SS method tend to be higher when compared to the BMD (bias + 55%) while the MTS method tends to yield MIC values that are lower in comparison to the BMD (bias − 18,8%). When examining *P. aeruginosa*, MIC values obtained from both the SS and MTS methods tend to be lower when compared to those obtained through BMD (bias: − 37,5% for both methods).

**Table 4:**
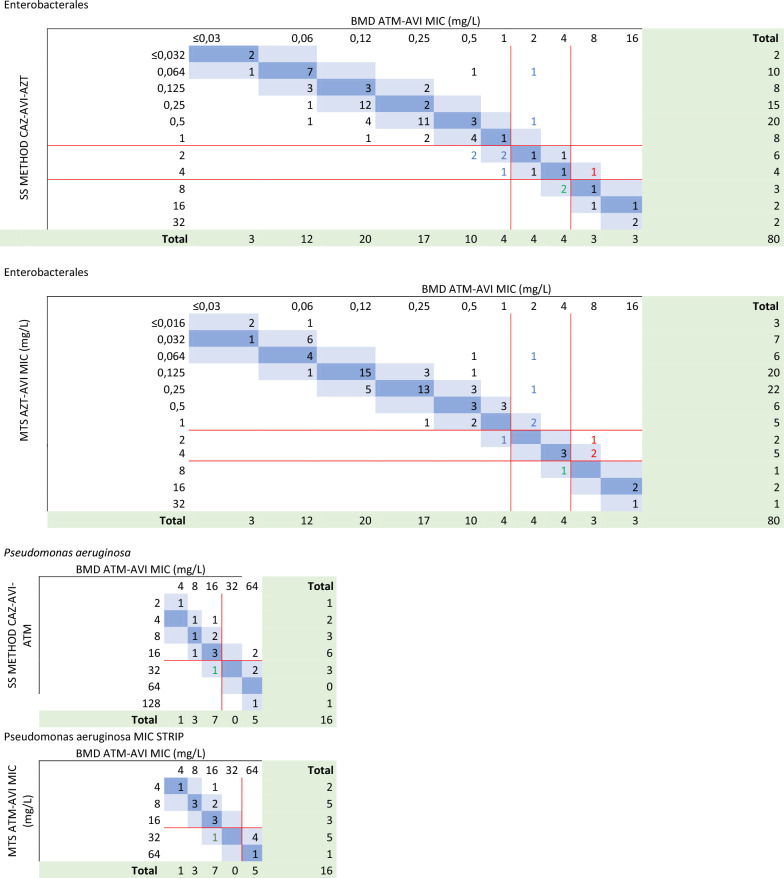
Broth microdilution and gradient diffusion tests (Etest strip stacking and MICStrip) MIC distributions of ATM in presence of AVI or of CAZ-AVI to assess the efficacy of the ATM-AVI combination

Discrepancies are numbers indicated by colors (blue: minor discrepancy, green: major discrepancy, red: very major discrepancy). Breakpoints are represented by red lines

### Disk elution method

Of the 96 results obtained with the DE method, CA reached 91% for *Enterobacterales* and 81% for *P. aeruginosa*, but a high number of VMD were observed for EB (n = 6; 8%) and for *P. aeruginosa* (n = 3; 19%) compared to other methods.

## Discussion

Carbapenem-resistant *Enterobacterales* (CRE), and particularly carbapenemase-producing *Enterobacterales* (CPE) are increasing worldwide and threaten the global public health. In Belgium, class B carbapenemases are produced currently by 40% of CPE strains with a significant increase in NDM-type CPE (from 11% in 2017 to 28% in 2022), which has become the second most prevalent carbapenemase after OXA-48 according to the National Reference Center for Antibiotic-Resistant Gram-Negative Bacilli [15].

The combined administration of ATM and CAZ-AVI has been used in various infections caused by MBL-producing *Enterobacterales*, including bloodstream, urinary, pulmonary, and joint infections [3, 16, 17]. ATM + CAZ-AVI proved to be the most effective among combinations against MBL-producing *Enterobacterales* [18] achieving a clinical resolution rate of 80% in infected patients according to a systematic review [13]. ATM + CAZ-AVI could serve as an interesting alternative strategy, awaiting the availability of ATM-AVI combination, in treating infections by MBL-producing *Enterobacterales* or strains that are resistant to cefiderocol or other last-line molecules. Data are more controversial regarding the efficacy of the combination against MBL-producing *P. aeruginosa* [19, 20].

Our study panel included 96 (100%) carbapenem resistant strains and 14 (15%) cefiderocol-resistant strains. 11 (11,4%) of those strains were resistant to the combination of ATM-AVI including 4 *E. coli* (all NDM producers), 2 *S. marcescens* (1 OXA-427 and 1 non carbapenemase) and 5 *P. aeruginosa* strains (3 non carbapenemase and 2 MBL). Our data of ATM-AVI resistance detected in *E. coli* isolates are in line with the literature describing similar observations of ATM-AVI resistance mainly in *E. coli* due to specific mechanisms involved such as PBP3 protein insertions (e.g., YRIN or YRIK) or CMY-42 β-lactamase (produced by two *E. coli* strains in our study) [21]. These ATM-AVI resistant strains, not often available and tested in other studies, allowed us to challenge evaluated methods. However, the small number of ATM-AVI-resistant strains (n = 6 in *Enterobacterales;* n = 5 in *P. aerguinosa*), prevents correctly assessing the ability of the tested methods in detecting such strains. Additionally, the absence of *K. pneumoniae* or *E. cloacae* ATM-AVI resistant strains hinders the assessment of the methods' performance in detecting such strains. This limitation was unbiased and reflected current epidemiological situation as depicted by one Belgian study showing no ATM-AVI resistance among *Enterobacterales* and one French study with few ATM-AVI resistant strains tested [14, 22]. Continuous surveillance should monitor potential significant emergence of ATM-AVI resistant strains.

The primary goal of this study was to evaluate the effects of the ATM-AVI combination and the accuracy of the different testing methods for ATM-AVI. Our results demonstrate that MIC values yielded by MTS method closely aligned with those obtained with BMD (EA > 90%). Other studies have also indicated the reliability of the MTS method [14, 22, 23]. Additionally, MTS method offers a technical convenience compared to the SS and DE methods. However, we highlighted a significant concern regarding the method's capability to detect ATM-AVI resistance, as only 1 out of 4 *E. coli* strain was accurately categorized as ATM-AVI-resistant. Moreover, we cannot assess the reliability of these methods for the detection of ATM-AVI resistance among *K. pneumoniae* and other *Enterobacterales* species given the lack of those strains in this study.

The SS method yielded fewer VMD than the MTS method for *Enterobacterales* but yielded more MD and minD. The complexity of the SS method, which involves manual operations to remove and replace from the agar plate the pre-incubated CAZ-AVI E-test strip by the ATM strip, may leads to inappropriate diffusion of avibactam in the agar plate or to bacterial contamination. Obviously, the SS method increases workload and costs compared to MTS ATM-AVI. Hence, if available, the MTS test would be the preferred choice for determining ATM-AVI MIC.

The DE method was first described by Khan et al. highlighting its affordability and accessibility in low-resource settings for screening rapidly the synergy between AVI and ATM [11]. In our study for *Enterobacterales*, DE method was reproducible and reached a good CA (90%), but high VMD (8%) was observed. It could therefore serve as a cheap alternative tool to test for synergy, although should be used with caution due to the risk of missing ATM-AZI resistance*.* However, this method is not appropriate for *P. aeruginosa* given the high rate of VMD (19%) and its low reproducibility (80%). We have not been able to provide explanations for the lower reproducibility observed for *P. aeruginosa* with the DE, as well as for the SS method. Ultimately, DE method has the drawback of not providing a MIC value, which may be important to guide clinical treatment against these difficult-to-treat resistant microorganisms.

Our study also examined whether the two molecules exhibited synergistic effects when combined and if a reduction in MIC values (≥ threefold) was observed compared to individual components. *Enterobacterales* showed an excellent activity recovery of 95% when exposed to the ATM-AVI combination. Our data are in line with other studies where all MBL-producing *Enterobacterales* were susceptible to the combination [22, 24, 25] underscoring the importance of considering this combination as empirical therapy for infections caused by these microorganisms. However, it had a limited activity against *P. aeruginosa,* as our result showed only 17% synergy. This observation supported by other studies is likely due to the coexistence of additional non-carbapenemase resistance mechanisms in *P. aeruginosa* [11, 22]. Therefore, the combination of ATM-AVI has very limited usefulness for the treatment of multidrug-resistant *P. aeruginosa* strains.

The strength of our study is that we used a collection of well-characterized and highly resistant clinical strains including the presence of a few, but yet significant number of ATM-AVI resistant strains, challenging the evaluated testing methods. This study has several limitations given its single-center design with a limited number of isolates (n = 96). Then, the absence of well-defined ATCC reference strains hinders the evaluation of result reproducibility. Finally, we believe the methods should be tested with more strains that exhibit ATM-AVI resistance to better evaluate the performance of resistance detection with the evolving epidemiology.

## Conclusion

The ATM-AVI association displayed excellent in vitro synergistic activity against extensively multidrug-resistant clinical *Enterobacterales* isolates in Belgium. Our data suggest that MTS method offers accurate ATM-AVI AST results on *Enterobacterales* strains, while the SS method might serve as better alternative then DE method in assessing the efficacy of ATM-AVI combination. Further investigation should ascertain methods’ ability to detect ATM-AVI resistance in *Enterobacterales*.

### Supplementary Information

Supplementary Material 1.

Supplementary Material 2.

## Data Availability

The datasets used and/or analysed during the current study are available from the corresponding author on reasonable request.
